# Investigation of Dietary Factors and Esophageal Cancer Knowledge: Comparison of Rural Residents in High- and Low-incidence Areas

**DOI:** 10.1038/s41598-018-23251-3

**Published:** 2018-03-20

**Authors:** Dong Tian, Shuai-Jia Mo, Lian-Kui Han, Liang Cheng, Heng Huang, Shuai Hao, Ye-Lan Guan, Kai-Yuan Jiang, Jing-Ya Deng, Hu-Hao Feng, Hong-Ying Wen, Mao-Yong Fu

**Affiliations:** 1 Department of Cardiothoracic Surgery, Affiliated Hospital of North Sichuan Medical College, Nanchong, 637000 China; 2 College of Basic Medicine, North Sichuan Medical College, Nanchong, 637000 China; 3 Department of Thoracic Surgery, Guizhou Provincial People’s Hospital, Guiyang, 550002 China

## Abstract

To compare the differences in dietary status and knowledge of esophageal cancer (EC) between residents of high- and low-incidence areas. We investigated dietary conditions and EC knowledge among residents in high- and low-EC incidence areas (Yanting and Qingzhen counties). Residents in Yanting consumed more pickled vegetables, salted meat and barbecued food (P < 0.05). Analysis of the past ten-year trend in Yanting consumed fresh vegetables/fruits, beans, sauerkraut, hot food, and barbecued food had gradually increased, and the trend was less than that in Qingzhen County. However, the gradual increasing trend in consumption of pickled vegetables, pickled meat, and spicy food over the past 10 years was greater (P < 0.05). Drinking water in Yanting County was healthier than that in Qingzhen County (P < 0.05). In terms of EC knowledge, the proportions of residents in Yanting who had a clear understanding, knowledge or had heard of EC or knew the common causes, primary symptoms, therapeutic measures, preventive measures, and government interventions for EC were all higher than in Qingzhen (P < 0.05). Residents in Yanting had greater EC knowledge but more harmful dietary habits than those in Qingzhen.

## Introduction

Esophageal cancer (EC) is a common malignant tumor of the digestive tract. EC-associated morbidity and mortality ranks eighth and sixth, respectively, among all cancers globally^1^. In China, there is a particularly high-EC incidence area in which the mortality and morbidity from EC rank fourth globally^2^. There are significant differences in the regional distribution of EC. The mortality rate in high-incidence areas is substantially higher than that in surrounding areas and shows an irregular concentric circular distribution, gradually decreasing in the surrounding areas^3^. In addition, there can be a 200- to 300-fold difference in the rates of EC between the high-incidence areas and low-incidence areas^4^. Meanwhile, EC lacks typical symptoms in the early stage of the disease. Most patients, especially those in rural areas, are in the middle-to-advanced stage and have missed the best chance for treatment^5^. Dietary factors, especially the salted and preserved food consumed in high-incidence areas of Asian countries, have been hypothesized to affect the risk of EC via different mechanisms, and many studies have demonstrated associations between dietary habits and EC morbidity^6–8^. However, epidemiological studies in Western countries have shown that smoking and alcohol consumption are important risk factors for EC in low-incidence areas^9,10^.

This survey was administered in Yanting County of Guizhou Province, which is a high-incidence area for EC, and in Qingzhen City, which is a low-incidence area. Yanting, a rural and one the most indigent counties in Sichuan Province, is located northeast of Chengdu City, at 105° latitude N and 31° longitude E. In addition to poverty, Yanting is also well known for its serious EC challenge. Qingzhen is a county in Guizhou Province (N24°30–29°13, E103°1–109°30, 1100 m above sea level, subtropical humid climate) in southwestern China, located in the Miaoling mountains. From 2006 to 2011, the age-adjusted incidence of EC in Yanting was 138.37/10^5^ for males and 68.04/10^5^ for females^11^ compared with the figures of 2.87/10^5^ in Guizhou Province^12^. Overall, the difference in the morbidity rate between the high- and low-incidence areas is obvious. Through a comparative analysis of the residents’ living conditions, dietary habits and EC knowledge, this study investigated the dietary habits of residents in high- and low-EC incidence areas to provide a reference for improvement of early diagnosis, early treatment and prevention of EC.

## Materials and Methods

### Study Design

The study was approved by the institutional ethics committee of the Affiliated Hospital of North Sichuan Medical College. Informed consent was obtained from all enrolled respondents. Detailed information about the dietary patterns and living environment in high- and low-EC incidence areas has been described previously^7,13^. All methods were applied according to the approved guidelines. This study recruited students from North Sichuan Medical College and Zunyi Medical College. After unified training, the investigators conducted face-to-face surveys using a self-designed questionnaire and recorded the survey results. The survey included general information and data regarding personal eating habits, family dietary status in the past year and the past 10 years, and knowledge of EC. In addition, the subject of salt taste preference was described in their daily diets. The degree of saltiness was depended on the median sodium intake, that was slightly salty <1 g/day, moderately salty 1–3 g/day and very salty >3 g/day, respectively as described in a previous study^14,15^. The eating speed was described as “rapid”, which indicated finishing their meal in 10 minutes; “usual”, which indicated finishing their meal in 30 minutes; and “slow”, which indicated finishing their meal in 1 hour or more. The food temperature was described as “hot” (over 60 °C), “cold” (less than 5 °C), and “moderate temperature” (5 °C–60 °C). Regarding the spiciness, the previous study showed that bell peppers are considered to have a Scoville rating of zero, that is, lacking any piquancy, whereas habanero peppers have a Scoville rating of 300,000. Pure capsaicin rates at 16 million Scoville units^16^. In our study, we designed the degree of spicy food with the Scoville rating of 300,000. That meant participants who intake the habanero peppers. A check signifies having eaten the food every day and was defined by the term “many”, an average intake per month of no more than 1 time was denoted “few”, and other values were defined as “moderate”.

### Respondents

A total of 987 residents of Yanting participated in the survey, and 920 valid questionnaires were completed. Hence, the effective response rate was 93.2%. Among the participants, 484 were male (52.6%) and 436 were female (47.4%), and the average age was 52.72 ± 17.68 years. A total of 330 rural residents of Qingzhen County, Guizhou Province participated in the survey, and 313 valid questionnaires were collected, yielding an effective response rate of 94.8%. Of these respondents, 163 were male (52.1%) and 150 were female (47.9%), and the average age was 55.96 ± 14.08 years. There was no significant difference in the sex or average age between the rural residents in the high- and low-incidence areas (P > 0.05).

### Statistical Analysis

Statistical analyses were performed using SPSS 22.0 software (SPSS, Inc., Chicago, IL, USA). The data are reported as the frequencies, means, and medians with percentages. A chi-square test was used to compare the categorical variables. For all statistical tests, a P-value < 0.05 was considered statistically significant.

## Results

### Dietary Status

With regarding to the personal dietary habits of the rural residents in the high-incidence area, the proportions of participants eating salty food, hard food, eating rapidly and hot food were 17.1%, 14.8%, 32.1%, and 19.1%, respectively, while the corresponding proportions of residents in the low-incidence area were 10.2%, 5.1%, 24.3% and 5.8%, respectively. The results of the Pearson chi-square tests for the dietary habits of the two groups of residents did not indicate a statistically significant difference (P > 0.05). The personal dietary habits of participants in the two counties were similar (Table 1).

**Table 1:** Personal dietary habits of the rural residents in the high- and low-EC incidence areas. The survey defines the tastes of the respondents by referring to the family members’ evaluations. The degree of saltiness was depended on the median sodium intake, that was slightly salty <1 g/day, moderately salty 1–3 g/day and very salty >3 g/day, respectively. The “eating speed” was described as “rapid”, which indicated finishing their meal in 10 minutes; “usual”, which indicated finishing their meal in “30 minutes”; or “slow”, which indicated finishing their meal in “1 hour or more”. The “food temperature” was described as “hot” (over 60 °C), “cold” (less than 5 °C), or “moderate temperature” (5 °C–60 °C).

Dietary habits	Yanting County of Sichuan Province (n = 920)	Qingzhen County of Guizhou Province (n = 313)	χ^2^	P
**Salt taste preference**			1.523	0.823
Very salty	157(17.1%)	32(10.2%)		
Moderately salty	375(40.8%)	195(62.3%)		
Slightly salty	388(42.2%)	86(27.5%)		
**Toughness of food**			6.496	0.370
Hard	136(14.8%)	16(5.1%)		
Moderate hardness	414(45.0%)	219(70.0%)		
Soft	369(40.1%)	78(24.9%)		
**Speed of eating**			6.917	0.329
Rapid	295(32.1%)	76(24.3%)		
Usual	395(42.9%)	143(45.7%)		
Slow	228(24.8%)	94(30.0%)		
**Temperature of food**				
Hot	176(19.1%)	18(5.8%)	2.544	0.637
Moderate temperature	612(66.5%)	281(89.8%)		
Cold	132(14.3%)	14(4.5%)		

In terms of the family dietary status of the rural residents in the high- and low-EC incidence areas in the past year, the residents in the high-incidence area usually ate fresh foods, such as vegetables/fruits and beans, accounting for 37.3% and 31.0% of the participants, respectively, and the corresponding proportions of participants in the low-incidence area were 37.4% and 25.2%, respectively, in the past year. There were no significant differences between the two groups (P > 0.05). In the past year, the proportions of pickled vegetables, pickled meat, and barbecued food that were consumed by the high- and low-incidence area residents were significantly different (P < 0.05). Residents in Yanting consumed more pickled vegetables, salted meat and barbecued food but similar amounts of fresh vegetables/fruits, beans, sauerkraut, hot food, and spicy food compared with those of Qingzhen County in the past year (Table 2).

**Table 2:** Family dietary status of the rural residents in the high- and low-EC incidence areas in the past year. We designed the degree of spicy food with the Scoville rating of 300,000. That meant participants who intake the habanero peppers. A check signifies having eaten the food every day and is defined by the term “many”, an average intake per month of no more than 1 time is denoted as “few”, and other values are defined as “moderate”. *P < 0.05 is statistically significant.

Dietary status	Yanting County of Sichuan Province (n = 920)	Qingzhen County of Guizhou Province (n = 313)	χ^2^	P
**Vegetables/fruits**			2.115	0.909
Many	343(37.3%)	117(37.4%)		
Moderate	200(21.7%)	114(36.4%)		
Few	377(41.0%)	82(26.2%)		
**Beans**			2.717	0.843
Many	285(31.0%)	79(25.2%)		
Moderate	231(25.1%)	136(43.5%)		
Few	404(43.9%)	98(31.3%)		
**Pickled vegetables**			12.881	0.045^*^
Many	306(33.3%)	8(2.6%)		
Moderate	172(18.7%)	70(22.4%)		
Few	442(48.0%)	235(75.1%)		
**Salted meat**			20.580	0.002^*^
Many	143(15.5%)	6(1.9%)		
Moderate	168(18.3%)	50(16.0%)		
Few	609 (66.2%)	257(82.1%)		
**Sauerkraut**			13.442	0.137
Many	197(21.4%)	74(23.6%)		
Moderate	178(19.3%)	122(39.0%)		
Few	545(59.2%)	117(37.4%)		
**Hot food**			6.334	0.387
Many	124(13.5%)	46(14.7%)		
Moderate	174(18.9%)	145(46.3%)		
Few	622(67.6%)	122(39.0%)		
**Spicy food**			6.394	0.380
Many	268(28.0%)	93(29.7%)		
Moderate	174(18.9%)	144(46.0%)		
Few	488(53.0%)	76(24.3%)		
**Barbecued food**			50.120	0.000^*^
Many	114(12.4%)	10(3.2%)		
Moderate	94(10.2%)	76(24.3%)		
Few	711(77.3%)	227(72.5%)		

A comparison of the high- and low-incidence areas suggests that the proportions of residents who gradually increased their consumption of vegetables/fruits, beans, pickled vegetables, pickled meat, sauerkraut, hot food, spicy food and barbecued food were 26.2% vs. 48.9%, 18.5% vs. 23.0%, 10.9% vs. 3.2%, 6.7% vs. 3.2%, 7.1% vs. 10.8%, 7.8% vs. 9.3%, 9.5% vs. 5.1% and 5.5% vs. 6.4%, respectively, in the past 10 years. There were statistically significant differences between the two groups (P < 0.05). The past ten-year trend in Yanting revealed that the proportions of residents who consumed fresh vegetables/fruits, beans, sauerkraut, hot food, and barbecued food with a gradually increasing trend were less than those of residents in Qingzhen County. However, the gradually increasing trends in consumption of pickled vegetables, pickled meat, and spicy food in Yanting County in the past 10 years were more than those in Qingzhen County (Table 3).

**Table 3:** Family dietary status of the rural residents in the high- and low-EC incidence areas in the past 10 years. Δ Each questionnaire surveyed the food intake of only one household over 10 years. *P < 0.05 is statistically significant.

Dietary status	Yanting County of Sichuan Province (n = 920)	Qingzhen City of Guizhou Province (n = 313)	χ^2^	P
**Vegetables/fruits**			19.066	0.004^*^
Invariant	530(57.6%)	144(46.0%)		
Gradual increase	241(26.2%)	153(48.9%)		
Gradual decrease	149(16.2%)	16(5.1%)		
**Beans**			17.213	0.009^*^
Invariant	611 (66.4%)	217(69.3%)		
Gradual increase	170 (18.5%)	72(23.0%)		
Gradual decrease	139(15.1%)	24(7.7%)		
**Pickled vegetables**			41.960	0.000^*^
Invariant	548 (59.5%)	207(66.1%)		
Gradual increase	100 (10.9%)	10(3.2%)		
Gradual decrease	272 (29.6%)	96(30.7%)		
**Pickled meat**			38.065	0.000^*^
Invariant	533(57.9%)	231(73.8%)		
Gradual increase	62(6.7%)	10(3.2%)		
Gradual decrease	325(35.3%)	72(23.0%)		
**Sauerkraut**			47.441	0.000^*^
Invariant	547(59.4%)	229(73.2%)		
Gradual increase	65(7.1%)	34(10.8%)		
Gradual decrease	308(33.5%)	50(16.0%)		
**Hot food**			32.428	0.000^*^
Invariant	550(59.8%)	238(76.0%)		
Gradual increase	72(7.8%)	29(9.3%)		
Gradual decrease	298(32.4%)	46(14.7%)		
**Spicy food**			17.179	0.009^*^
Invariant	532(57.8%)	245(78.3%)		
Gradual increase	87(9.5%)	16(5.1%)		
Gradual decrease	301(32.7%)	52(16.6%)		
**Barbecued food**			38.582	0.000^*^
Invariant	565(61.4%)	221(67.4%)		
Gradual increase	51(5.5%)	20(6.4%)		
Gradual decrease	304(33.1%)	82(26.2%)		

In the high-EC incidence area, the main source of drinking water was tap water which in the permissible value of Drinking Water Quality Standards in China, accounting for 72.5% of the residents. In the low-incidence area, the main source of drinking water was also tap water, accounting for 59.1% of the residents. Compared with the drinking water of the residents in the high-EC incidence area, that of the residents in the low-incidence area used less tap water. The drinking water sources of the two areas were significantly different (P < 0.05) (Table 4).

**Table 4:** Family drinking water sources of the rural residents in the high- and low-EC incidence areas. ^Δ^In the permissible value of Drinking Water Quality Standards in China. *P < 0.05 is statistically significant.

	Yanting County of Sichuan Province (n = 920)	Qingzhen City of Guizhou Province (n = 313)	χ^2^	P
**Drinking water sources**			111.075	0.000^*^
Tap water^Δ^	667(72.5%)	185(59.1%)		
Deep water in a pressurized well	101(11.0%)	10(3.2%)		
Shallow water in a pressurized well	54(5.9%)	14(4.5%)		
Deep water in a water injection well	56(6.1%)	12(3.8%)		
Shallow water in a water injection well	3(0.3%)	6(1.9%)		
Spring in a water injection well	39(4.2%)	86(27.5%)		

The EC knowledge among rural residents in the high- and low-incidence areas was also surveyed in our study. The percentages of residents in the high- vs. low-incidence areas who had heard of EC and those with a clear understanding of the common causes, main symptoms, therapeutic measures, preventive measures, and government interventions were 17.0% vs. 4.5%, 12.1% vs. 4.5%, 13.5% vs. 3.8%, 7.8% vs. 1.9%, 8.9 vs. 1.9%, and 6.9% vs. 1.9%, respectively. All proportions were higher in the high-incidence are than in the low-incidence area. There were statistically significant differences between the two areas (P < 0.05) (Table 5).

**Table 5:** Personal knowledge of EC among the rural residents in the high- and low-incidence areas. ^Δ^The common causes, main symptoms, treatment measures and preventive measures for EC were found in the 8th edition of the AJCC & UICC (2017). EC, Esophageal cancer. *P < 0.05 is statistically significant.

Contents of the survey	Yanting County of Sichuan Province (n = 920)	Qingzhen City of Guizhou Province (n = 313)	χ^2^	P
**Ever heard of EC**			18.761	0.027^*^
Never heard of	239(26.0%)	176(56.2%)		
Know of	525(57.1%)	123(39.3%)		
Clear understanding of	156(17.0%)	14(4.5%)		
**Common causes of EC**			20.267	0.016^*^
Never heard of	613(66.6%)	279(89.1%)		
Know of	196(21.3%)	20(6.4%)		
Clear understanding of	111(12.1%)	14(4.5%)		
**Main symptoms of EC**			11.048	0.042^*^
Never heard of	605(65.8%)	276(88.2%)		
Know of	191(20.8%)	25(8.0%)		
Clear understanding of	124(13.5%)	12(3.8%)		
**Therapeutic measures for EC**			20.733	0.014^*^
Never heard of	629(75.2%)	297(97.9%)		
Know of	156(17.0%)	10(3.2%)		
Clear understanding of	77(7.8%)	6(1.9%)		
**Preventive measures for EC**			10.282	0.028^*^
Never heard of	704(76.5%)	297(87.9%)		
Know of	135(14.7%)	32(10.2%)		
Clear understanding of	81(8.9%)	6(1.9%)		
**Government interventions**			11.548	0.031^*^
Never heard of	749(81.4%)	275(87.9%)		
Know of	107(11.6%)	32(10.2%)		
Clear understanding of	64(6.9%)	6(1.9%)		

## Discussion

EC is the result of a combination of causes and is not associated with only a single factor^17^. Because of the coordination effect between EC and factors such as the living environment, dietary habits, smoking history, alcohol drinking, low social and economic status, poor standard of living and inadequate knowledge related to EC^18^, the combination of carcinogenic factors and cellular genetic factors may result in gradual accumulation of genetic mutations in cells responsible for malignant tumors^7,13^.

Many epidemiological studies have shown there is an important relationship between EC occurrence and dietary status^7,19–21^. Residents with EC in a high-incidence area have diets characterized by hot food, dry food, pickled vegetables, barbecued food, and spicy food, which can cause chronic physical and chemical damage to the esophageal mucosa, increasing its susceptibility to carcinogenic factors and carcinogenesis^13,22^. In our study, the proportions of participants eating salty food, hard food, eating rapidly, and hot food in the high-incidence area were higher than those in the low-incidence are. However, there was no significant difference in personal eating habits between the rural residents in the two areas (P > 0.05). This finding may be due to the dietary habits of residents in both areas.

Pickled vegetables, pickles, sauerkraut and barbecued food contain N-nitroso-compounds (NOCs), which have been found in animal models to induce malignant tumors, including EC^23,24^. Epidemiological studies have also shown that the NOC content in food in high-incidence areas is remarkably higher than that in the low-incidence areas of China^23^. There is a significant positive correlation between the NOC level in food and EC morbidity^25^. NOCs may play an important role in EC carcinogenesis in China. In terms of family diet in the past year, the proportions of residents who consumed high amounts of pickled vegetables, pickles, and barbecued food in the high-incidence area were significantly higher than those in the low-incidence area (P < 0.05). Additionally, in the past 10 years, the consumption of pickled vegetables and pickled meat, which contain NOCs, was also significantly higher in the high-incidence area than in the low-incidence area (P < 0.05). Eating more vegetables/fruits and beans is a protective factor against EC because they increase the intake of anti-carcinogenic substances and reduce the incidence of EC^7,20,21^. In our study, there were no significant differences in the consumption of vegetables/fruits and beans between the two areas in the past year (P > 0.05). However, in the past 10 years, the proportion of vegetable/fruit intake has gradually increased, and the consumption of beans by residents was significantly lower in the high-incidence area than in the low-incidence area (P < 0.05). In the past year and in the past 10 years, the high-incidence area residents tended to consume more harmful foods. Our results showed that although many protective measures were performed in Yanting, changing the dietary habits of the residents in the area is difficult, which might explain why Yanting remains a high-incidence area after more than twenty years of preventative measures^1^.

Many studies have demonstrated the relationship between drinking water sources and EC^19,26^. The type and quality of drinking water are related to the intake of water with “three nitrogen” content in high-EC incidence areas^26–28^. Well water is considered to be contaminated with nitrite nitrogen that reacts with amines *in vivo* to form N-nitroso compounds, which are the causative factors for EC^28^. Low quality drinking water has been confirmed to be a risk factor in China in previous studies^22,29^. In our study, approximately 72.5% and 59.1% of residents in the high- and low-EC incidence areas, respectively, used tap water, which contains less contaminants, as the main source of drinking water. The drinking water sources in the two areas were significantly different (P < 0.05). The water quality in the high-incidence area was demonstrably better than that in the low-incidence area. This finding is related to government measures to implement projects that improved tap water quality, which were considered to be protective factor and had been popular in Yanting County.

Many previous studies have shown a 5-year survival rate of more than 95% for patients with early-stage EC^30–32^. However, EC lacks typical symptoms at the early stage of the disease, and it can take 2–3 years from the time of onset to the manifestation of symptoms. Once admitted to the hospital, most patients are in the advanced stage of disease and have thus missed the best time for treatment^32^. Prevention and treatment of EC in the high-incidence area of Yanting has been performed for many years^11^. However, knowledge of the prevention and control of EC was still unclear in high-incidence areas. No study concerning the differences in EC knowledge between high- and low-incidence areas has been conducted previously. In our study, we surveyed the knowledge of EC in both high- and low-incidence areas. Our survey demonstrated that there were statistically significant differences in whether residents had ever heard about EC and their understanding of the common causes, main symptoms, therapeutic measures, preventive measures, and government intervention in the high- versus low-incidence areas (P < 0.05). Residents in the high-incidence area had better knowledge of EC than those in the low-incidence area in these aspects. This finding may be related to propaganda from the government and disease propagandists who popularized the knowledge of EC in high-incidence areas. Besides, it may because participants from high incidence area may know more knowledge of EC from other EC patients than that of low incidence area of EC. They may also know that from their family member or neighbors. To decrease the morbidity and increase the diagnostic rate of EC, early diagnosis and treatment should be performed in high-incidence areas. Popularization of EC knowledge is an important way to promote residents’ awareness of preventive and curative measures in high-incidence areas^33,34^. Therefore, improving public awareness of EC should be helpful for reducing the mortality and morbidity of EC.

In summary, the dietary habits and knowledge of EC in high- and low-incidence areas were different in our survey. Residents in the high-incidence area had more harmful dietary habits than those in the low-incidence area, although many preventative measures and control policies have been performed during these years in the high-incidence area. In addition, residents in the high-incidence area had greater knowledge of EC than those in the low-incidence area. However, the prevalence of knowledge regarding EC prevention and control still needs to be strengthened.
