# Author Correction: Investigation of Dietary Factors and Esophageal Cancer Knowledge: Comparison of Rural Residents in High- and Low-incidence Areas

**DOI:** 10.1038/s41598-018-26886-4

**Published:** 2018-05-31

**Authors:** Dong Tian, Shuai-Jia Mo, Lian-Kui Han, Liang Cheng, Heng Huang, Shuai Hao, Ye-Lan Guan, Kai-Yuan Jiang, Jing-Ya Deng, Hu-Hao Feng, Hong-Ying Wen, Mao-Yong Fu

**Affiliations:** 1 Department of Cardiothoracic Surgery, Affiliated Hospital of North Sichuan Medical College, Nanchong, 637000 China; 2 College of Basic Medicine, North Sichuan Medical College, Nanchong, 637000 China; 3 Department of Thoracic Surgery, Guizhou Provincial People’s Hospital, Guiyang, 550002 China

Correction to: *Scientific Reports* 10.1038/s41598-018-23251-3, published online 20 March 2018

This Article contains errors in the Introduction section.

“This survey was administered in Yanting County of Guizhou Province, which is a high-incidence area for EC, and in Qingzhen City, which is a low-incidence area. Yanting, a rural and one the most indigent counties in Sichuan Province, is located northeast of Chengdu City, at 105° latitude N and 31° longitude E.”

should read:

“This survey was administered in Yanting County of Sichuan Province, which is a high-incidence area for EC, and in Qingzhen City of Guizhou Province, which is a low-incidence area. Yanting, a rural and one the most indigent counties in Sichuan Province, is located northeast of Chengdu City, at 31° latitude N and 105° longitude E.”

